# *Borna* [bor′nә] *disease virus*

**DOI:** 10.3201/eid1509.999999

**Published:** 2009-09

Borna disease virus was named after the town of Borna in Saxony, southeastern Germany, where in 1885 many horses in a German cavalry regiment died of a fatal neurologic disease. The ill horses exhibited abnormal behavior—running about excitedly, walking into walls, being unable to chew food. A similar disease had been observed in horses, sheep, and cattle for more than 100 years. The causative agent was later found to be a negative-stranded RNA virus, which may also be a human pathogen.

